# [Retracted] Curculigoside attenuates myocardial ischemia-reperfusion injury by inhibiting the opening of the mitochondrial permeability transition pore

**DOI:** 10.3892/ijmm.2025.5716

**Published:** 2025-12-15

**Authors:** Yanbing Zhao, Yuxuan Guo, Yuqiong Chen, Shuang Liu, Nan Wu, Dalin Jia

Int J Mol Med 45: 1514-1524, 2020; DOI: 10.3892/ijmm.2020.4513

Following the publication of this paper, a concerned reader drew to the Editor's attention that a pair of the fluorescence microscopic images shown in Fig. 2A on p. 1518 were strikingly similar to data which had already been accepted for publication in the journal *The Anatolian Journal of Cardiology* written by different authors, although the same department and research institute were held in common. Upon performing an independent analysis of the data in this paper in the Editorial Office, it also came to light that flow cytometric data in Fig. 2B had already been submitted for publication in another paper to the journal *Drug Design, Development and Therapy* that featured some of the same authors, although the experimental conditions in the two papers were reported to be different.

Owing to the fact that the contentious flow cytometric and fluorescence microscopic data in the above article had apparently already been submitted for publication elsewhere, the Editor of *International Journal of Molecular Medicine* has decided that this paper should be retracted from the Journal. The authors were asked for an explanation to account for these concerns, but the Editorial Office did not receive a satisfactory reply. The Editor apologizes to the readership for any inconvenience caused.

